# Combined effect of the smartphone addiction and physical activity on the depressive symptoms in secondary school students: a cross sectional study in Shanghai, China

**DOI:** 10.3389/fpsyt.2024.1473752

**Published:** 2025-01-03

**Authors:** Rui Yang, Shuoyuan Tan, Gulqihra Abdukerima, Ting Lu, Chen Chen, Lixin Song, Bing Ji, Yipeng Lv, Jianwei Shi

**Affiliations:** ^1^ School of Public Health, Shanghai Jiao Tong University School of Medicine, Shanghai, China; ^2^ Shanghai Jiao Tong University of Medicine, Shanghai, China; ^3^ Shanghai Jing’an District Jiangning Road Community Health Service Center, Shanghai, China; ^4^ Department of General Practice, Yangpu Hospital, Tongji University School of Medicine, Shanghai, China; ^5^ Department of Social Medicine and Health Management, School of Public Health, Shanghai Jiao Tong University School of Medicine, Shanghai, China

**Keywords:** depressive symptoms, smartphone addiction, physical activity, adolescents, China

## Abstract

**Objective:**

This study aims to investigate the prevalence of smartphone addiction, physical activity levels, and depressive symptoms among secondary school students, and to analyze the combined impact of smartphone addiction and physical activity on depressive symptoms.

**Methods:**

A cluster sampling method was employed in two secondary schools in the Jing’an District of Shanghai, China. Univariate analysis was used to compare the prevalence of depressive symptoms across different demographic characteristics. Logistic regression was utilized to examine the associations between smartphone addiction, physical activity, and their combined effect on depressive symptoms.

**Results:**

A total of 1,316 respondents participated in the study, with reported prevalence rates of depressive symptoms (36.2%), smartphone addiction (19.2%), and insufficient physical activity (23.3%). Risk factors for depressive symptoms included being a non-only child (OR=1.421, 95% CI: 1.090-1.853, P=0.009), inadequate sleep duration (OR=2.722, 95% CI: 2.070-3.578, P<0.001) and smartphone addiction (OR=2.173, 95% CI: 1.621-2.913, P < 0.001). Adolescents with smartphone addiction were significantly more likely to report depressive symptoms compared to those without (OR=2.173, 95% CI: 1.621-2.913, P < 0.001). Joint analysis indicated that combined smartphone addiction and insufficient physical activity significantly increased the risk of depressive symptoms (OR=2.781, 95% CI: 1.627-4.753, P < 0.001).

**Conclusion:**

The study identified a high prevalence of severe smartphone addiction, insufficient physical activity, and elevated rates of depressive symptoms among secondary school students. Smartphone addiction and inadequate physical activity were associated with increased likelihood of depressive symptoms. Moreover, higher levels of physical activity appeared to mitigate the adverse impact of smartphone addiction on depressive symptoms.

## 1 Introduction

In recent decades, as wireless network technology has become more pervasive and smartphone functionalities have advanced, there has been a noticeable trend towards younger individuals using smartphones. This trend has brought to light the issue of smartphone addiction among adolescents. Smartphone addiction is characterized by an inability to control phone use (1), leading to physiological issues like headaches and difficulty concentrating, as well as emotional challenges such as anxiety, depression, and reduced social adaptability (2, 3).Research has indicated that the global reporting rate of smartphone addiction was 28.3%, with variations observed across different cultural backgrounds, ranging from 10% to 31% (4). Eastern cultural backgrounds tend to report higher rates of smartphone addiction compared to Western cultures (5).

Another issue associated with smartphone addiction is the insufficient physical activity observed among adolescents globally (6). This lack of activity hinders the attainment of normal growth and development standards and has emerged as a significant public health concern. Research highlighted by Hallal showed that over 80% of adolescents worldwide failed to achieve the recommended 60 minutes of moderate-to-vigorous physical activity daily (7).

These aforementioned issues may be closely related to depression among adolescents. Depression, as one of the most prevalent mental disorders, affects approximately 350 million people of various ages worldwide. It is anticipated that by 2030, depression will constitute the largest global disease burden, posing a significant public health challenge to the international community (8). Adolescent depression is often considered to be a precursor of the adult depression and can lead to enduring social and psychological harm (9). Adolescents are particularly vulnerable to depression due to genetic, social, and environmental factors, resulting in a higher incidence compared to other age groups. Recent studies indicated a strong correlation between smartphone addiction, insufficient physical activity and psychological symptoms such as anxiety and depression. Tang et al. (2018) observed that adolescents and young adults addicted to mobile phones exhibited increased mental vulnerability, heightening their risk of depression (10). Additionally, individuals in Asian countries with smartphone addiction tended to have higher rates of depression compared to those in the United States. This suggested that cultural factors and environmental context should also not be ignored when exploring the impact of smartphone addiction and physical inactivity on adolescent mental health. Research among British adolescents aged 11-14 revealed a negative association between physical activity and depressive symptoms: increasing weekly exercise by 60 minutes reduced the risk of developing depressive symptoms by 8% (11). Furthermore, Kim et al. (2020) demonstrated that moderate-to-vigorous physical activity moderates the relationship between screen time and depressive symptoms (12). Studies on Chinese college students by Feng et al. (2014) and Wu et al. (2015) similarly found that those with both smartphone addiction and insufficient physical activity had a higher likelihood and severity of depressive symptoms (13, 14). However, there remains a paucity of research investigating the combined impact of smartphone addiction and insufficient physical activity on depressive symptoms, particularly in adolescents.

This study examined the prevalence of smartphone addiction, physical activity level, and depressive symptoms among secondary school students. It analyzed how smartphone addiction and insufficient physical activity impacted depressive symptoms. Additionally, it explored whether increased physical activity could mitigate the negative effects of smartphone addiction on depressive symptoms. The aim of this study is to provide insights for effective interventions targeting adolescent depression.

## 2 Materials and methods

### 2.1 Participants

A cross-sectional survey was conducted in Shanghai from November to December 2023. Using a cluster sampling method, the research team collaborated with two secondary schools in Jing ‘an District, Shanghai, an economically developed area in China. Participants included students from grades 6, 7,and 8 in junior secondary school, and grades 10 and 11 in senior secondary school. Students in grades 9 and 12 were excluded due to the transition pressures associated with higher education. After obtaining the consent of all participants and their legal guardians, all eligible participants independently completed the questionnaire including demographic information. In addition, variables for student height and weight were obtained by physical examination of the students. Gender, age, type of school, type of family structure, one-child status, BMI, and sleep duration were all included as control variables in the regression of smartphone addiction, physical activity, and depressive symptoms. A total of 1,327 students participated, with 1,316 providing valid responses (effective response rate: 99.2%).

### 2.2 Ethical approval

The research adhered to local legislation and institutional requirements.

### 2.3 Measures

#### 2.3.1 Smartphone addiction

Smartphone addiction was assessed using the Smartphone Addiction Scale Short-Version (SAS-SV) modified by Kwon, based on the Smartphone Addiction Scale (SAS) (15). Xiang et al. (16)revised this scale in Chinese in 2019, and conducted a reliability and validity test in adolescents, and the results showed that the scale had good reliability and validity in adolescents. Mu et al. also used the Chinese version of the scale to investigate smartphone addiction in their study, and verified Cronbach’s α =0.92 (17).The scale consists of 10 items rated on a 6-point Likert scale (1 = strongly disagree to 6 = strongly agree), yielding a total score ranging from 10 to 60. Scores > 32 indicated smartphone addiction. The Cronbach’s α coefficient for reliability was 0.920.

#### 2.3.2 Physical activity level

The physical activity level was assessed using the Chinese University of Hong Kong: Physical Activity Rating for Children and Youth (CUHK-PARCY) scale. The CUHK-PARCY scale was adapted based on the Jackson Activity Coding and the Godin-Shephard Activity Questionnaire (18, 19). It encompasses a range of 11 physical activity levels, from complete inactivity (0 points) to vigorous exercise (10 points), demonstrating robust validity and aggregate reliability. In this study, scores on the CUHK-PARCY questionnaire were categorized as follows: scores of 0-2 indicated insufficient physical activity, scores of 3-6 denoted a moderate physical activity level, and scores of 7-10 represented a high physical activity level (20).

#### 2.3.3 Depressive symptoms

Depressive symptoms were assessed by using the Self-rating Depression Scale (SDS) developed by Zung, consisting of 20 items rated on a 4-point scale (1 = none or rare to 4 = most or all the time) (21). The scale includes both positive and reverse-scored items, with the total scores summed to obtain the raw score. The standard score is derived by multiplying the total raw score by 1.25, resulting in an integer score. A standard score higher than 53 indicates the presence of depressive symptoms. In this study, the Cronbach’s α coefficient for the SDS was calculated to be 0.886.

### 2.4 Statistical analysis

Statistical analysis was performed using IBM SPSS 26.0 software. Differences in depressive symptoms among adolescents with various characteristics were assessed using the χ2 test. Binary logistic regression was employed for both univariate and multivariate analyses to investigate the independent effects of smartphone addiction and physical activity on adolescent depressive symptoms, calculating Odds Ratios (OR) across different groups. To explore the combined effects of smartphone addiction and physical activity on depressive symptoms, the two behaviors were categorized into 6 groups (2 smartphone addiction groups × 3 physical activity level groups). The non-smartphone addiction, high physical activity group served as the reference to compare the risk of depressive symptoms across different groups of smartphone addiction and physical activity level.

## 3 Results

### 3.1 Basic information of study subjects

**Table 1** presented data on the 1,316 participants, with 623 (47.3%) identified as female and 693 (52.7%) as male. Depressive symptoms were reported in 477 participants, constituting 36.2% of the total sample. Smartphone addiction was observed in 253 participants (19.2%). Regarding physical activity levels, the distribution from highest to lowest was moderate (39.1%), high (37.7%), and insufficient (23.3%) physical activity groups.

**Table 1:** Demographics of the secondary school students (n=1316).

Characteristics	Total n (%)	Depressive symptoms		χ²	P value
		Yes (n=477)	No (n=839)		
Gender				1.651	0.199
Female	623 (47.3)	237 (38.0)	386 (62.0)		
Male	693 (52.7)	240 (34.6)	453 (65.4)		
Age (years)				0.481	0.488
≥15	654 (49.7)	231 (35.3)	423 (64.7)		
<15	662 (50.3)	246 (37.2)	416 (62.8)		
School type				0.439	0.508
Senior secondary school	656 (49.8)	232 (35.4)	424 (64.6)		
Junior secondary school	660 (50.2)	245 (37.1)	415 (62.9)		
Family structure type				0.834	0.361
Non-nuclear family	328 (24.9)	112 (34.1)	216 (65.9)		
Nuclear family	988 (75.1)	365 (36.9)	623 (63.1)		
The only child				9.631	0.002
No	335 (25.5)	145 (43.3)	190 (56.7)		
Yes	981 (74.5)	332 (33.8)	649 (66.2)		
BMI				4.95	0.084
Obesity	203 (15.4)	84 (41.4)	119 (58.6)		
Overweight	263 (20.0)	103 (39.2)	160 (60.8)		
Normal	850 (64.6)	290 (34.1)	560 (65.9)		
Sleep duration (h)				51.583	<0.001
Insufficient (<7h)	431 (32.8)	215 (49.9)	216 (50.1)		
Sufficient (≥7h)	885 (67.2)	262 (29.6)	623 (70.4)		
Smartphone addiction				34.386	<0.001
Addiction	253 (19.2)	132 (52.2)	121 (47.8)		
Non-addiction	1063 (80.8)	345 (32.5)	718 (667.5)		
Physical activity level				6.798	0.033
Insufficient physical activity	306 (23.3)	127 (41.5)	179 (58.5)		
Moderate physical activity level	514 (39.1)	189 (36.8)	325 (63.2)		
High physical activity level	496 (37.7)	161 (32.5)	335 (67.5)		

### 3.2 Influencing factors of depressive symptoms in secondary school students

**Table 2** illustrated that as obesity levels increase, so did the risk of developing depressive symptoms. Participants in the overweight group (OR=1.263, 95% CI: 0.934-1.706, P=0.129) and obesity group (OR=1.434, 95% CI: 1.028-2.001, P=0.034) showed a higher likelihood of depressive symptoms compared to the normal weight group. Smartphone addiction (OR=2.173, 95%CI: 1.621-2.913, P<0.001), non-only child status (OR=1.421, 95%CI:1.090-1.853, P=0.009), and insufficient sleep duration (OR=2.722, 95%CI:2.070-3.578, P<0.001) were identified as risk factors for depressive symptoms in secondary school students.

**Table 2:** Logistic regression analysis of factors influencing depressive symptoms (n=1,316).

Characteristics	β	OR (95%CI)	P value
Gender			
Female	0.084	1.087 (0.847-1.395)	0.510
Male			
Age (years)			
≥15	-0.325	0.722 (0.130-4.025)	0.710
<15			
School type			
Senior secondary school	-0.265	0.767 (0.137-4.293)	0.763
Junior secondary school			
Family structure type			
Non-nuclear family	-0.141	0.868 (0.660-1.143)	0.315
Nuclear family			
The only child			
No	0.351	1.421 (1.090-1.853)	0.009
Yes			
BMI			
Obesity	0.361	1.434 (1.028-2.001)	0.034
Overweight	0.233	1.263 (0.934-1.706)	0.129
Normal			
Sleep duration (h)			
Insufficient (<7)	1.001	2.722 (2.070-3.578)	<0.001
Sufficient (≥7)			
Smartphone addiction			
Addiction	0.776	2.173 (1.621-2.913)	<0.001
Non-addiction			
Physical activity level			
Insufficient physical activity	0.290	1.337 (0.968-1.846)	0.078
Moderate physical activity level	0.183	1.201 (0.909-1.586)	0.198
High physical activity level			

### 3.3 Combined effect of smartphone addiction and physical activity on depressive symptoms

**Figure 1:**
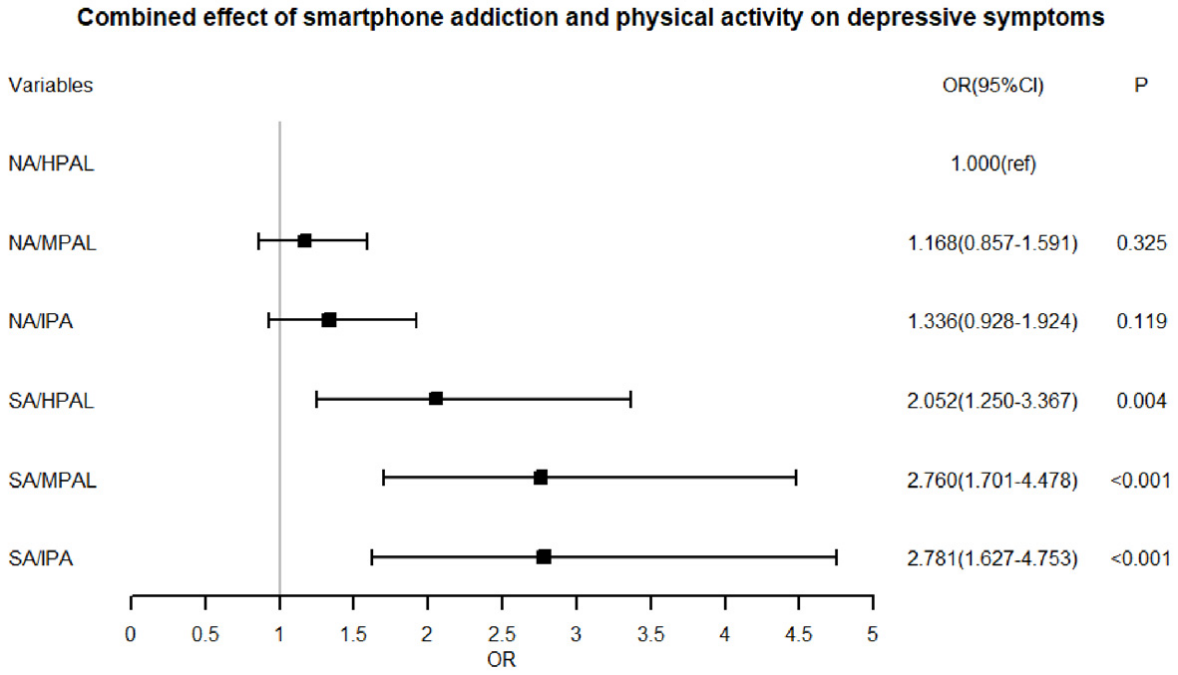
Combined effect of smartphone addiction and physical activity on depressive symptoms. Corresponding to Model 2 in **Table 3**, adjustments were made for gender, age (years), school type, family structure type, only-child status, and sleep duration.

**Table 3** revealed differences in reporting rates of depressive symptoms across various levels of physical activity and smartphone addiction groups. Using the non-smartphone addiction and high physical activity level group as references: Non-smartphone addiction, moderate physical activity level group: OR=1.168, 95%CI: 0.857-1.591, P=0.325; Non-smartphone addiction, insufficient physical activity group: OR=1.336, 95%CI: 0.928-1.924, P=0.119; Smartphone addiction, high physical activity level group: OR=2.052, 95%CI: 1.250-3.367, P=0.004; Smartphone addiction, moderate physical activity level group: OR=2.760, 95%CI: 1.701-4.478, P <0.001; Smartphone addiction, insufficient physical activity group: OR=2.781, 95%CI: 1.627-4.753, P <0.001. When no smartphone addiction was present, there were no statistically significant differences in the reporting rates of depressive symptoms among any physical activity level groups (P > 0.05, **Figure 1**).

**Table 3:** Combined effects of smartphone addiction and physical activity on depressive symptoms.

Smartphone addiction	Physical activity	Number of depressive symptoms/total number	Model 1		Model 2	
			OR (95%CI)	P value	OR (95%CI)	*P* value
NA	HPAL	118/410	1.000		1.000	
NA	MPAL	140/421	1.233 (0.918-1.655)	0.164	1.168 (0.857-1.591)	0.325
NA	IPA	87/232	1.485 (1.056-2.088)	0.023	1.336 (0.928-1.924)	0.119
SA	HPAR	43/86	2.475 (1.541-3.974)	<0.001	2.052 (1.250-3.367)	0.004
SA	MPAR	49/93	2.756 (1.740-4.364)	<0.001	2.760 (1.701-4.478)	<0.001
SA	IPA	40/74	2.911 (1.757-4.823)	<0.001	2.781 (1.627-4.753)	<0.001

NA, Non-addiction; SA, Smartphone addiction; HPAL, High physical activity level; MPAL, Moderate physical activity level; IPA, Insufficient physical activity. Model 1 was unadjusted for confounding variables. Model 2 was adjusted for gender, age (years), learning phase, family structure type, only-child status, and sleep duration.

## 4 Discussion

During adolescence, individuals undergo rapid psychological development, making them particularly susceptible to external influences that can result in various degrees of psychological issues. In this study, the prevalence of depressive symptoms among participants was 36.2%, significantly higher than both the 8% reported globally in previous studies on adolescent depression and the 26.3% prevalence observed among Chinese adolescents (22, 23). Despite potential influences from factors such as questionnaire tools, economic development, and social culture, this study consistently highlighted a high incidence of depressive symptoms among adolescents, underscoring the critical need for enhanced mental health interventions and improved adolescent mental health care. In addition, the study further revealed that adolescents who were non-only children or experience insufficient sleep duration demonstrated notably higher reporting rates of depressive symptoms, corroborating findings from prior research (24, 25).Future attention should prioritize the mental health needs of non-only children and adolescents with inadequate sleep duration, recognizing these factors as significant predictors of depression.

With the proliferation of smartphone technology, adolescents increasingly engage in activities such as communication, studying, and entertainment via smartphones. However, due to their ongoing development of self-control mechanisms, adolescents often struggle with smartphone addiction. This study found a 19.2% prevalence of smartphone addiction among participants. Smartphone addiction not only negatively impacts physical health, such as reduced vision and poorer sleep quality, but it can also contribute to various psychological issues including anxiety, depression, and reduced social adaptability (2, 26). This phenomenon poses significant public health challenges globally (27). Moreover, the study identified smartphone addiction as an independent risk factor for depressive symptoms, with significantly higher reporting rates among those addicted compared to non-addicted peers, aligning with previous research by Park et al. (2018) (28). The social replacement hypothesis posits that excessive smartphone use displaces real social interactions, potentially leading to negative emotions like depression (26, 29). Additionally, disruptions to circadian rhythms caused by smartphone use can impair neuroendocrine function, further predisposing adolescents to depressive symptoms (30). These findings highlight the urgent need for interventions targeting adolescent smartphone addiction. Strategies may include promoting face-to-face social interactions and offering educational resources to cultivate healthy smartphone use habits (31–33).

Notably, smartphone addiction often coexists with insufficient physical activity. The study reported prevalence rates of 37.7% for high physical activity, 39.1% for moderate physical activity, and 23.3% for insufficient physical activity among participants, reflecting a significant deviation from the UN’s recommended standards for adolescent physical activity. Prolonged physical inactivity not only contributes to physical ailments such as overweight and obesity but also serves as a key factor in the development of psychological disorders, including depressive symptoms (11). Thus, enhancing adolescent physical activity levels holds considerable importance for promoting both physical and mental well-being. Therefore, we recommend that physical activity promotion be included in public health strategies for adolescents. Schools and communities can work together to provide more accessible opportunities for sports and outdoor activities, which can both prevent physical health problems and improve mental health (34, 35).

This study also examined the combined effect of smartphone addiction and physical activity on the incidence of depressive symptoms. Compared to the students experiencing either smartphone addiction or insufficient physical activity alone, those affected by both conditions reported a higher prevalence of depressive symptoms. This finding was supported by previous research conducted by Liu et al. (2019) and Xie et al. (2019) (36, 37). When adolescents exhibited smartphone addiction, their likelihood of experiencing depressive symptoms was markedly elevated irrespective of their level of physical activity. This suggests that smartphone addiction exerts a more pronounced negative influence than insufficient physical activity, although higher levels of physical activity may partially mitigate the detrimental effects of smartphone addiction on adolescents’ depressive symptoms. The underlying rationale may stem from the possibility that smartphone addiction contributes to reduced physical activity levels, thereby nullifying the protective benefits of physical activity on mental health (38). In sight of this, interventions should not target smartphone addiction or physical inactivity in isolation, but also address both factors simultaneously, and policymakers and educators should develop comprehensive interventions to encourage reduced smartphone use and increased physical activity, providing practical solutions for incorporating healthy behaviors into adolescents’ daily lives. For example, schools and healthcare providers should implement targeted programs to educate adolescents about the importance of balancing smartphone use and physical activity, while encouraging daily physical activity, such as team sports or outdoor recreation, to help adolescents adopt a healthier daily lifestyle. In addition, parents should be educated about the signs of smartphone addiction and its impact on mental health, and provided with strategies regarding setting healthy boundaries in balancing smartphone use. By combining these strategies in both school and home environments, it is hoped that a more supportive environment can be created for adolescents to reduce their risk of developing or exacerbating depressive symptoms.

This study not only addresses a theoretical gap by providing a comprehensive analysis of smartphone addiction and physical activity, but also offers significant practical implications. The analysis of the combined effects of smartphone addiction and physical activity on depressive symptoms provides new insights for policymakers, urging the integration of support from all sectors of society to develop more effective, multifaceted intervention strategies aimed at addressing the increasing prevalence of adolescent depression. Furthermore, this study identifies additional key factors contributing to adolescent depression, including being a non-only child and sleep deprivation. These findings underscore the necessity for individualized interventions targeting these specific risk factors. Policymakers and mental health professionals can utilize these insights to design more targeted, evidence-based strategies to better mitigate the onset of adolescent depression. Additionally, the exploration of the interaction between various behavioral and demographic factors in the development of depression emphasizes the importance of a holistic approach to adolescent mental health. Several limitations of this study also need to be addressed. Firstly, the data were cross-sectional, precluding definitive conclusions regarding the causal relationships among smartphone addiction, physical activity, and depressive symptoms. Secondly, the study sample was confined to two secondary schools selected through cluster sampling, potentially limiting its generalizability to the broader adolescent population. Lastly, apart from height and weight, all variables were self-reported, which introduces the possibility of recall bias and reporting inaccuracies. Future studies should incorporate more objective assessment tools and adopt prospective methodologies to enhance data accuracy.

## 5 Conclusion

In summary, the prevalence of depressive symptoms among adolescents is substantial, with both smartphone addiction and insufficient physical activity posing significant challenges. A combined effect of smartphone addiction and insufficient physical activity on depressive symptoms was observed. Controlling smartphone addiction was found to be crucial in reducing the occurrence and progression of depressive symptoms in adolescents. Increasing levels of physical activity could partially mitigate the adverse effects of smartphone addiction on depressive symptoms. Therefore, comprehensive interventions targeting both smartphone addiction and physical activity are crucial for effectively managing the incidence and progression of depressive symptoms in adolescents.

## Data Availability

The raw data supporting the conclusions of this article will be made available by the authors, without undue reservation.
